# Sharing of photobionts in sympatric populations of *Thamnolia* and *Cetraria* lichens: evidence from high-throughput sequencing

**DOI:** 10.1038/s41598-018-22470-y

**Published:** 2018-03-13

**Authors:** Ioana Onuț-Brännström, Mitchell Benjamin, Douglas G. Scofield, Starri Heiðmarsson, Martin G. I. Andersson, Eva S. Lindström, Hanna Johannesson

**Affiliations:** 1 Systematic Biology, Department of Organismal Biology, Uppsala University, Uppsala, Sweden; 2 Evolutionary Biology, Department of Ecology and Genetics, Uppsala University, Uppsala, Sweden; 3 Uppsala Multidisciplinary Center for Advanced Computational Science (UPPMAX), Uppsala University, Uppsala, Sweden; 4 Icelandic Institute of Natural History, Akureyri Division, Borgir Nordurslod, Iceland; 5 Limnology, Department of Ecology and Genetics, Uppsala University, Uppsala, Sweden

## Abstract

In this study, we explored the diversity of green algal symbionts (photobionts) in sympatric populations of the cosmopolitan lichen-forming fungi *Thamnolia* and *Cetraria*. We sequenced with both Sanger and Ion Torrent High-Throughput Sequencing technologies the photobiont ITS-region of 30 lichen thalli from two islands: Iceland and Öland. While Sanger recovered just one photobiont genotype from each thallus, the Ion Torrent data recovered 10–18 OTUs for each pool of 5 lichen thalli, suggesting that individual lichens can contain heterogeneous photobiont populations. Both methods showed evidence for photobiont sharing between *Thamnolia* and *Cetraria* on Iceland. In contrast, our data suggest that on Öland the two mycobionts associate with distinct photobiont communities, with few shared OTUs revealed by Ion Torrent sequencing. Furthermore, by comparing our sequences with public data, we identified closely related photobionts from geographically distant localities. Taken together, we suggest that the photobiont composition in *Thamnolia* and *Cetraria* results from both photobiont-mycobiont codispersal and local acquisition during mycobiont establishment and/or lichen growth. We hypothesize that this is a successful strategy for lichens to be flexible in the use of the most adapted photobiont for the environment.

## Introduction

Symbiosis is any type of close and long-term biological interaction between at least two different organisms. Across the Tree of Life, there are numerous examples of independently arisen symbiotic interactions with similar ecological and/or morphological outcomes^1^ among which lichens and reef corals being representative examples. These long-lived symbiotic entities are formed by heterotrophic organisms, fungi or animals, which acquire their carbon from the autotrophic microbes. Lichens, in contrast to the climate dependent corals^2^, are successful colonizers of moderate to extreme biomes and climatic zones^3^ and outperform vascular plants in both biodiversity and biomass in these areas^4^. Still, the slow growth of lichens and the impracticability of experimental work have resulted in a largely unknown life cycle and a limited understanding of the mechanism of adaptation to the environment. The focus of this study is the dynamics of the association between the heterotrophic fungus (*i*.*e*., the mycobiont) and the autotrophic microbe (the photobiont) that constitute lichens.

It is widely accepted that the mycobiont, with its net-like mycelium, provides the lichen its shape and structure, while the photobiont contributes with the carbon source^5^. The mycobiont also offers the matrix of the symbiotic vegetative propagules (soredia, isidia or minute thallus fragments), where symbiotic partners are embedded and dispersed as one functional unit to form a new lichen individual^6,7^. In addition, many lichenized fungi reproduce sexually by producing meiospores that can disperse and form new lichens by acquiring compatible photobionts and other symbiotic partners from external sources^5^. The fact that the most common genera of green algae photobionts (*Trebouxia* and *Pseudotrebouxia*) rarely form free-living colonies as their aerial relatives^7^), make lichens’ source of new photobionts puzzling^8^. It has been hypothesized that free-living mycobionts are ephemeral, and an advantageous strategy for lichens that disperse by meiospores is to use as immediate source photobiont cells from already established lichens, dying lichens or vegetative propagules^9,10^. Accordingly, lichen lineages do not constitute cospeciating symbiotic fungi and algae; rather, horizontal transmission (*i*.*e*., switching) of photobionts between different fungal lineages frequently occurs^11,12^. Photobiont switching seems to be an ubiquitous phenomenon in lichens and appears to play a vital role in lichens adaptation to environment^8,13^. Furthermore, this is not a lichen specific phenomenon, but was also shown in reef corals, which can survive elevate temperatures and recover from bleaching episodes if they succeed to associate with a specifically adapted photobiont lineage^2,14^. Although the photobiont adaptive hypothesis was only occasionally experimentally tested in lichens^15,16^, several studies have shown a strong correlation between the photobiont genotype and ecology or geography of the lichen^4,17,18^. Additionally, it was shown that photobiont genotypes can be shared by diverse mycobionts at the same location^17,19–30^. All of these findings advocate that lichen forming fungi associate with local adapted photobiont strains that will be selected and hence increase in frequency within population^19^. Lichens photobionts do not show a significant population structure between localities, but identical photobiont genotypes were shown to be encountered across wide geographic ranges^4,31^, suggesting that the vegetative dispersing mode of lichens is an important contributor on photobiont distribution^23,32^.

At the same time, it is yet not clarified how many photobiont genotypes individual lichen thalli can contain. Traditional Sanger sequencing of PCR products containing amplicons of ITS-region has been used for the molecular identification of photobionts in lichen thalli (e.g.^31^) and this methods is not optimal for detecting multiple photobionts in a tissue. However, the results from studies using different methodological approaches, including High-Throughput Sequencing (HTS), challenge the dogma of single photobiont genotypes in a lichen thallus, and suggest the presence of a heterogeneous photobiont population within a lichen individual^15,33–37^.

The aims of this study were (1) to explore the diversity of photobionts in lichen symbioses in nature, and (2) to compare the sensitivity of Sanger and IonTorrent HTS technologies for detecting photobiont genotypes in lichens. For this purpose, we used a simple study design to investigate photobionts of two cosmopolitan, ecological similar but distantly related^38^ lichenized fungal genera, *Thamnolia* Ach. ex Schaer. and *Cetraria* Ach. We investigated the diversity of photobionts in sympatric populations of *Thamnolia* and *Cetraria* from two geographic localities (Iceland and Öland). We also investigated whether the retrieved local photobiont genotypes can be encountered in *Thamnolia* and *Cetraria* individuals from other parts of the world. Our HTS data suggest that multiple photobiont genotypes coexist within lichen thalli and revealed sharing of photobionts in sympatric population. This study increases our understanding of the nature of symbioses formed by heterotrophs and autotrophs, and may spur other researchers to use HTS methodology to examine the photobiont community of lichens.

## Materials and Methods

### Study system

*Thamnolia* and *Cetraria* were selected since they are often found growing together in nature. Furthermore, there are previous reports of symbiont flexibility in *Thamnolia*^31,39^ and photobiont switching has previously been correlated with the colonization of climatically different regions in *Cetraria*^4^. Both *Thamnolia* and *Cetraria* can be encountered in high alpine and tundra environments, but *Cetraria* species have a wider distribution and are also found in the boreal forest or even warm, arid habitats^4^. Both genera appear to mainly reproduce through vegetative propagules, via lichen fragments as in *Thamnolia*^40–42^ or through diaspores (isidia: small outgrowths of lichen thallus) produced by *Cetraria*^4^. In addition, both genera produce fungal spores that will need to re-associate with local photobionts to form new lichens^4,43^.

### Field sampling and morphological identification

**Figure 1:**
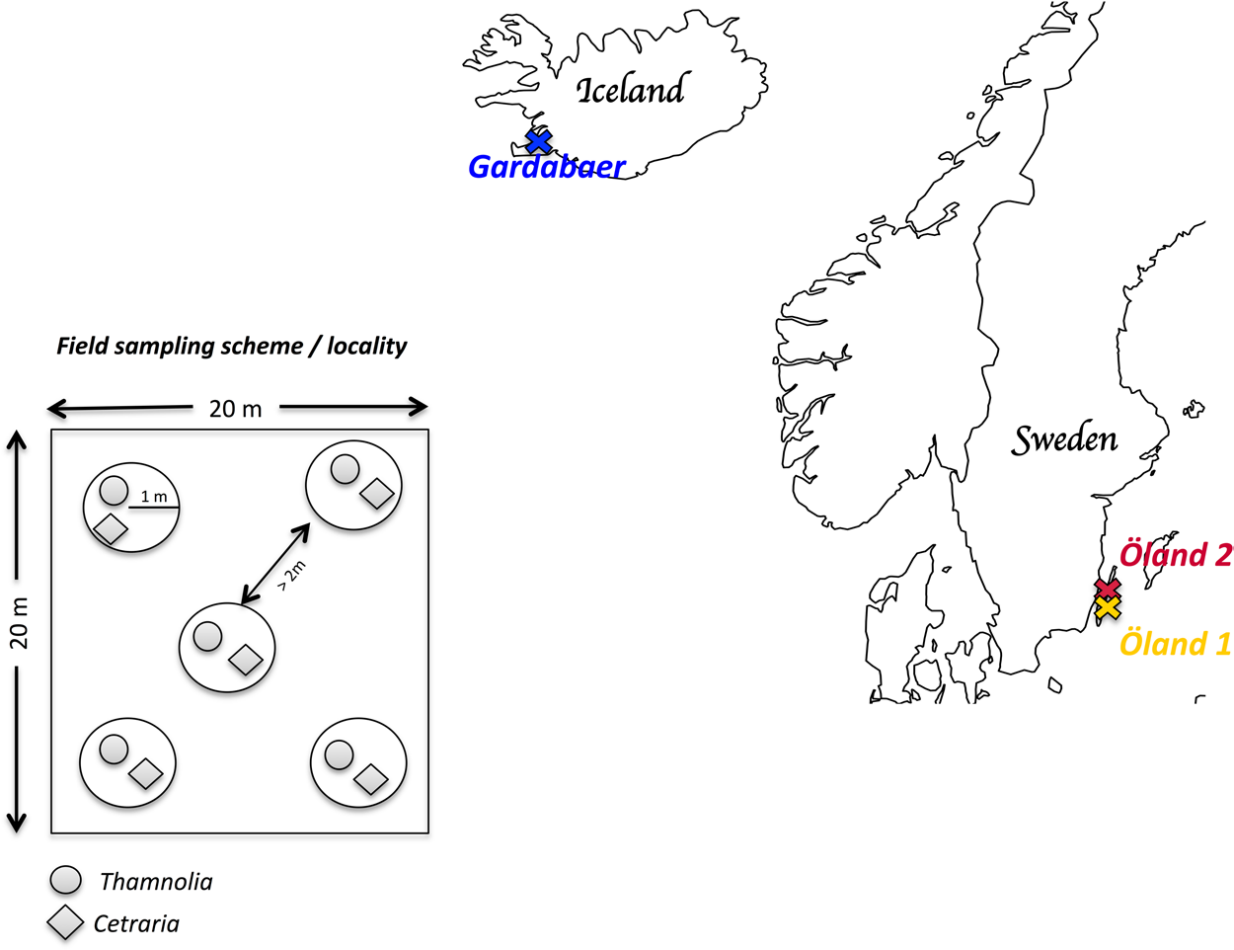
Investigated localities and sampling scheme. The map with the three investigated sites: one on Iceland (Gardabaer) and two in Sweden (Öland 1 and Öland 2) and the field sampling protocol. Within an area of 400 m^2^, five circles with one-meter in radius and with at least two meters distance between each other were selected. Within each circle, *Thamnolia* and *Cetraria* specimens (individual lichen thalli) were collected and for further analyses, we used one specimen of each genus from each circle.

The lichen samples used for this study were collected from two islands representing two different habitats: the melur of Iceland (sandy, gravelly hills with sparse vegetation)^44^ and the alvar of Öland, Sweden (limestone plain with very thin soil layer)^45^. On Iceland one site was sampled (Gardabaer), while on Öland two sites were sampled (Öland 1 and Öland 2) (Fig. 1, Table 1). There was a noticeable difference in the soil color at the different alvar sites; while Öland 1 had a yellowish color, indicating goethite enrichment and an increased moisture availability, Öland 2 had a reddish color, which indicates a good soil drainage and the presence of unhydrated iron oxides^46^. The field sampling was performed as follows: within an area of 400 m^2^, five circles with one-meter radius and with at least two meters distance between each other were selected (Fig. 1). Within each circle, *Thamnolia* and *Cetraria* specimens (individual lichen thalli) were collected and identified as *Thamnolia subuliformis* (Ehrh.) W.L. Culb., *Cetraria aculeata* sensu lato (Schreb.) Fr. or *Cetraria islandica* (L.) Ach by using morphological characters. For further analyses, we used one specimen of each genus from each circle (Fig. 1, Table 1).

**Table 1:** Lichen samples used in this study. All samples were deposited to The Museum of Evolution Herbarium, Uppsala University, Sweden (herbarium vouchers). GenBank accession numbers for the ITS sequences amplified through Sanger sequencing from the mycobiont and photobiont are given for each sample. For each locality lichen tissue from all five samples from one genus (Thamnolia or Cetraria) were pooled together for the Ion Torrent sequencing (pooled samples). ENA accession numbers for the amplicons generated by IonTorrent are shown for each pool.

Site	habitat	Geographic description	Lichen species	Individual sample ID	Pool ID	ENA accession number	GenBank Sanger ITS		
							herbarium voucher	mycobiont	photobiont
Iceland	melur	Iceland, Vestur-Island, Garðabær municipality, S of Vífilsstaðaháls	*Thamnolia subuliformis*	T.sub1_Ice	1	ERR2307093	L-851027	MG250331	MG250287
			*Thamnolia subuliformis*	T.sub2_Ice			L-851028	MG250332	MG250288
			*Thamnolia subuliformis*	T.sub3_Ice			L-851029	MG250333	MG250289
			*Thamnolia subuliformis*	T.sub4_Ice			L-851030	MG250334	MG250290
			*Thamnolia subuliformis*	T.sub5_Ice			L-851031	MG250335	MG250291
			*Cetraria aculeata* s. lat	C.acu1_Ice	2	ERR2307094	L-851034	MG250317	MG250302
			*Cetraria aculeata* s. lat	C.acu2_Ice			L-851035	MG250318	MG250303
			*Cetraria aculeata* s. lat	C.acu3_Ice			L-851036	MG250319	MG250304
			*Cetraria islandica*	C.isl4_Ice			L-851032	MG250320	MG250305
			*Cetraria aculeata* s. lat	C.acu5_Ice			L-851037	MG250321	MG250306
Öland 1	alvar	Sweden, Öland Island, Stora Alvaret, between S. Bårby and Alby	*Thamnolia subuliformis*	T.sub6_Oel1	3	ERR2307095	L-851013	MG250336	MG250292
			*Thamnolia subuliformis*	T.sub7_Oel1			L-851014	MG250337	MG250293
			*Thamnolia subuliformis*	T.sub8_Oel1			L-851015	MG250338	MG250294
			*Thamnolia subuliformis*	T.sub9_Oel1			L-851016	MG250339	MG250295
			*Thamnolia subuliformis*	T.sub10_Oel1			L-851012	MG250340	MG250296
			*Cetraria aculeata* s. lat	C.acu6_Oel1	4	ERR2307096	L-851017	MG250322	MG250307
			*Cetraria islandica*	C.isl7_Oel1			L-851022	MG250323	MG250308
			*Cetraria islandica*	C.isl8_Oel1			L-851018	MG250324	MG250309
			*Cetraria aculeata* s. lat	C.acu9_Oel1			L-851019	MG250325	MG250310
			*Cetraria aculeata* s. lat	C.acu10_Oel1			L-851020	MG250326	MG250311
Öland 2	alvar	Sweden, Öland Island, Stora Alvaret, between S. Möckleby and Torngård	*Thamnolia subuliformis*	T.sub11_Oel2	5	ERR2307097	L-851023	MG250341	MG250297
			*Thamnolia subuliformis*	T.sub12_Oel2			L-851008	MG250342	MG250298
			*Thamnolia subuliformis*	T.sub13_Oel2			L-851009	MG250343	MG250299
			*Thamnolia subuliformis*	T.sub14_Oel2			L-851011	MG250344	MG250300
			*Thamnolia subuliformis*	T.sub15_Oel2			L-851010	MG250345	MG250301
			*Cetraria islandica*	C.isl11_Oel2	6	ERR2307098	L-851021	MG250327	MG250312
			*Cetraria aculeata* s. lat	C.acu12_Oel2			L-851024	MG250328	MG250313
			*Cetraria islandica*	C.isl13_Oel2			L-851025	MG250329	MG250314
			*Cetraria islandica*	C.isl14_Oel2			L-851026	MG250330	MG250315
			*Cetraria islandica*	C.isl15_Oel2			L-851033	*	MG250316

*Unsuccessful PCR reaction.

### DNA extraction

For each selected specimen, we divided the lichen tissue in two halves: one half to be analysed using Sanger sequencing and one using HTS based Ion Torrent technology (see Supplementary Fig. S1). For Sanger sequencing, DNA extractions were separately performed on all 30 specimens listed in Table 1. For Ion Torrent sequencing we pooled for each locality the five specimens of *Thamnolia* into one sample, and the five specimens of *Cetraria* in another sample (Table 1, see Supplementary Fig. S1). We used 5 mg tissue of each specimen for DNA extraction. For both sequencing technologies, DNA was isolated with DNeasy Plant Mini Kit (Qiagen, GmbH, Hilden, Germany).

### Sanger sequencing of the mycobiont and photobiont ITS regions

PCR reaction was executed with the Thermo-Scientific Green Phusion High-Fidelity DNA polymerase kit (Thermo Fisher Scientific Inc. Waltham, MA, USA), according to the manufacturer’s instructions. From each of the 30 samples of Table 1, the ITS region of the mycobiont was amplified with the general primers ITS1F and ITS4, and the ITS region of the photobiont was amplified using the general primer pair ITS1T and ITS4T (Table S1). The PCR products were cleaned of unwanted dNTPs and primers using Exonuclease I (Thermo Fisher Scientific, SE) and Shrimp Alkaline Phosphatase (Affymetrix). The BigDye Teminator v 3.1 Cycle Sequencing and BigDye XTerminator Purification Kits (Thermo Fisher Scientific Inc. Waltham, MA, USA) were used to prepare the sample to be sequenced with Sanger technology by the ABI 3730 XL machine of Evolutionary Biology Centre, Uppsala University. All PCR products were sequenced from both directions using the same primers that were used for PCR amplification. The chromatograms of the sequences were visually inspected and curated by using the program Sequencher v5.2.3 (Gene Codes Corporation, Ann Arbor, MI, USA) and Geneious R9 (http://www.geneious.com)^47^. Sequencher was used to identify heterozygous sites in the sequences generated by Sanger technology: when two peaks with over 80% area overlap were encountered in a sequence, the site was identified as heterozygous, and the IUPAC ambiguity code was used for that particular site.

### Verification of mycobionts in the pooled samples

To verify that we did not have any cross-contamination between samples of *Thamnolia* and *Cetraria* in the six pooled samples of lichens used for Ion Torrent sequencing (Table 1), we used genus specific primers to verify the mycobiont. As control, we mixed DNA from the two genera in ratios of 1:9, 1:5 and 1:1 and performed two rounds of amplification on all of these DNA samples by using either *Thamnolia* and *Cetraria* fungal ITS specific primers designed for this study (Table S1).

### Ion Torrent sequencing of the ITS1 region of photobionts

PCR was executed with the Thermo-Scientific Phusion High-Fidelity DNA polymerase kit to amplify a part of the algal ITS1 region of maximum size 350 pb, using barcoded primers specifically designed for this study by the bioinformatics support from the Ion Torrent Sequencing center at SciLifeLab in Uppsala (Table S1). We also used two negative controls in which the template DNA was replaced by sterile water. Each sample for PCR (including the negative control) had its own barcoded primer combination. Gel electrophoresis was used to confirm the presence of PCR products of the expected size in the samples with DNA and the absence of PCR products in the negative controls. We used DNA Clean and Concentrator kit (Zymo Research, Orange, CA) to clean the amplicons from unwanted primers and dNTPs. The PCR product concentrations were measured with Qubit fluorometric quantification (Life Technologies), where after a single solution with all barcoded amplicons represented in equal amounts, and the two negative controls, was created. The library preparations with fusion primers were performed according to the manufacturers instructions. Uppsala SciLifeLab sequenced the pooled amplicons samples on an Ion Torrent PGM platform (Ion Torrent Xpress) using the 314^TM^ Chip KIT and 400 bp reads. The raw reads are deposited to the European Nucleotide Archive (ENA) (see Table 1 for the ENA accession numbers).

### Delineating and quantifying operational taxonomic units from Ion Torrent data

The Ion Torrent reads were processed to trim adapter and low-quality sequences using TorrentSuite BaseCaller 4.0-6/76303. To verify adapter removal, we constructed an adapter sequence list using Ion Torrent documentation, choosing A and P1 adapters along with the common prefix of barcode adapters, and scanned FastQ-format read sequences for each sample using FastQC (http://www.bioinformatics.babraham.ac.uk/projects/fastqc/). None of the candidate adapter sequences were detected.

To cluster amplicon sequences into operational taxonomic units (OTUs) for photobiont delineation and quantification, we used USEARCH 8.1.1861^48^ following recommended practices for Roche 454 reads (http://drive5.com/usearch/manual/upp_454.html, accessed 2016/06/01), which have an error profile similar to data obtained by Ion Torrent sequencing. All OTU-related trimming, clustering and quantification steps were performed using USEARCH. We explored a variety of read trim lengths and expected-error counts using the –fastq_eestats2 option, and selected trimming to 200 bp with 1.5 expected errors per read; this allowed us to retain sufficient length for each OTU to make comparisons with our Sanger sequences and sequences downloaded from databases. FastQ reads for all samples were pooled, dereplicated, and clustered into OTUs using default settings, with the additional constraint of requiring each OTU to be supported by at least two reads (−minsize 2). Thus, OTUs were formed using reads from all samples analysed together, with singleton OTUs excluded. Abundances of OTUs within each pooled set of reads were then estimated via global alignment of sampled-tagged reads, with minimum identity 97%. This method ensures continuity of OTUs across samples, enables formation of OTUs that might only have sufficient read support in aggregate while avoiding formation of singleton OTUs, and allows for OTUs to be quantified within samples in which they are rare. The script implementing this pipeline together with the candidate adapter sequences used during the scan with FastQC are available at https://github.com/douglasgscofield/pubs/tree/master/Brannstrom-et-al-1.

To example the dependence of OTU richness estimates on read counts, which varied somewhat among samples (mean 36740 ± 8300 [s.d.], range 22486–43110), we determined rarefaction curves using estimated_observation_richness.py in QIIME 1.9.1^49,50^. Richness estimates differed little throughout the range of observed read counts (see Supplementary Fig. S2), so we made no further correction to OTU diversity estimates.

### Taxonomic verification of mycobionts and photobionts

To assign the taxonomic identity of the mycobiont for each specimen identified as *Thamnolia* or *Cetraria* based on morphological characters, we compared the newly generated mycobiont and photobiont ITS sequences, both Sanger and Ion Torrent data, to sequences available at the GenBank nucleotide database^51^. We identified the best hits based on query coverage, sequence identity and e-value. The best hits that also had a species name assigned, using additional supporting evidence, were retrieved from GenBank. Subsequently, we employed a phylogenetic approach (see below) to investigate the relationship among the retrieved GenBank sequences and our newly generated data.

### Phylogenetic analyses

For each independent analysis, the sequences were aligned with mafft^52^ and trimmed in AliView v1.18^53^. All sequence alignments and the tree files resulted from this study analyses were deposited to https://github.com/douglasgscofield/pubs/tree/master/Brannstrom-et-al-1.

We chose the most appropriate strategy for phylogenetic analyses based on the presence of heterozygous sites and the amount of variable sites within each alignment.

Since we identified heterozygous sites in the data from photobiont Sanger sequences of the complete ITS region, the program RRHS version 1.0.0.2^54^ was used to randomly draw (with replacement) 1000 phased alignments of the trimmed alignment of the 30 photobiont Sanger sequences. For each of the 1000 phased alignments, phylogenetic trees were generated using the maximum likelihood (ML) with garli 2.01^55^ by running for each alignment five independent searches. Kimura 80 was selected as model for sequence evolution using the jModelTest. The 1000 trees with the best likelihood score were compiled in one phylogeny that represents the 50% majority-rule consensus tree using the program SumTrees^56^.

*Thamnolia* and *Cetraria* are highly divergent mycobiont genera from Lecanoromycetes^38^. Therefore we performed a separate phylogeny for samples of each genus based on the mycobiont Sanger sequences of the complete ITS region of our newly generated data together with GenBank references of sequences. For *Thamnolia* we chose representative ITS sequences for the previously discovered lineages A, B and C^31^ with the following accession numbers: KY550180, KY550083, KY550130, KY550098, KY550079, KY550170, KY550163, KY550143, KY550153, KY550127, KY550194. For *Cetraria* we used ITS sequences previously identified as *C*. *aculeata*, *C*. *muricata* and *C*. *islandica* (Accession numbers: AF228302, AF192409, EF373589, EF373567, AF228299). Both sets of sequences were aligned in AliView with mafft, and TrNef + G was chosen with JModel Test as sequence evolution model. Maximum likelihood phylogenies were performed with garli 2.01 together with 500 bootstrap trees. SumTrees script was used to place the bootstrap values on the branches.

The alignment of our newly generated photobiont sequences (ITS1 region) amplified with Sanger and Ion Torrent and the GenBank references (Table S2) presented many ambiguously aligned regions. To be able to use all sequence information, the software BAli-Phy 2.3.8^57^, which simultaneously estimates the alignment and the phylogeny using Bayesian inference, was used. We used JModel test^58^ to define HKY with a gamma distribution as the nucleotide substitution model for the photobiont phylogeny. Five MCMC chains with 20000 iterations each were run. The convergence of chains was diagnosed with the program Tracer^59^ and with “bp-analyze.pl” script of BAli-Phy 2.3.8. The same script was used to calculate the posterior probabilities of the 50% majority-rule consensus tree of all the MCMC chains, after 2000 iterations were discarded as burn in. All phylogenies were visualized in FigTree 1.4.2 (http://tree.bio.ed.ac.uk/software/figtree/).

### Sharing of photobiont OTUs

Non-metric multidimensional scaling (NMDS) was used to visualize similarities among the lichen samples with regard to photobiont diversity. The analyses were performed in R employing the function metaMDS in Vegan^60^ using the Bray-Curtis dissimilarity index. Before this analysis, the data sets were resampled (using the function rrarefy from Vegan) to an equal number of sequences per sample (22486). The NMDS analysis was run for 2 dimensions and 2000 iterations and the plot was generated in R using ggplot2^61^. The script used for this analysis is available at https://github.com/douglasgscofield/pubs/tree/master/Brannstrom-et-al-1.

The number of shared and specific OTUs between *Thamnolia* and *Cetraria* for each of the three localities was visualized as Venn diagrams that were constructed using the following web tool: http://bioinformatics.psb.ugent.be/webtools/Venn/.

The relative quantities of photobiont OTUs shared at each sampling location between *Thamnolia* and *Cetraria* were visualized by a hierarchical cluster analysis. The OTU read counts per sample were normalized to range from 0–1 within each sample, and were then clustered by similarity separately among OTUs and among site/genus pairs using hclust and dist in R. Distances were calculated via the Canberra method, as this permitted clustering to remain sensitive to similarities among more rare OTUs, unlike Euclidean distances, while retaining quantitative sensitivity unlike Boolean presence/absence distances. For display, OTU quantities were scaled by their cube-root, and colored by clade membership as determined by the phylogenetic analysis described above. The R script implementing this analysis is available at https://github.com/douglasgscofield/pubs/tree/master/Brannstrom-et-al-1.

### Data Availability

All the herbarium vouchers are deposited in UPS (The Museum of Evolution Herbarium – Uppsala, Sweden) and their herbarium accessions numbers can be found in Table 1. All the DNA sequences are deposited in GenBank and ENA. The accession numbers can be found in Table 1 (for the Sanger sequences) and Table 2 (for the Ion Torrent sequences). DNA sequences alignments are uploaded online in https://github.com/douglasgscofield/pubs/tree/master/Brannstrom-et-al-1.

**Table 2:** Photobiont OTUs identified by Ion Torrent sequencing technology. The number of reads for each OTU in each pooled lichen and negative control (NC) sample is shown. For all samples the reads were pooled, dereplicated, and clustered into OTUs using the constraint of requiring each OTU to be supported by at least two reads (−minsize 2). A total number of reads obtained for each sample is summed up at the bottom of each column.

OTUs ID	Iceland		Öland 1		Öland 2		NC	GenBank numbers
	Thamnolia	Cetraria	Thamnolia	Cetraria	Thamnolia	Cetraria		
OTU 1	38	9	41830	2	42020	0	1	MG372066
OTU 2	520	41200	1	8974	0	4301	0	MG372067
OTU 3	0	44	0	15220	2	33480	0	MG372068
OTU 4	0	0	0	132	0	218	0	MG372069
OTU 5	0	0	0	6209	0	0	0	MG372070
OTU 6	0	0	0	47	0	115	0	MG372071
OTU 7	0	0	0	393	0	821	0	MG372072
OTU 8	0	0	0	12	0	16	0	MG372073
OTU 9	0	0	75	0	61	0	0	MG372074
OTU 10	0	0	24	0	28	0	0	MG372075
OTU 11	0	0	0	11	0	27	0	MG372076
OTU 12	1	0	108	0	158	0	0	MG372077
OTU 13	4	346	0	68	0	43	0	MG372078
OTU 14	0	0	0	8	0	0	0	MG372079
OTU 15	0	0	9	0	13	0	0	MG372080
OTU 16	0	0	5	0	3	0	0	MG372081
OTU 17	124	2	0	3	0	0	0	MG372082
OTU 18	23	5	0	0	0	1	0	MG372083
OTU 19	0	0	5	0	12	0	0	MG372084
OTU 20	404	1	0	2	0	0	0	MG372085
OTU 21	0	0	0	1	0	3	0	MG372086
OTU 22	0	0	7	0	3	0	0	MG372087
OTU 23	10	11	0	6	0	1	0	MG372088
OTU 24	21360	1	0	0	0	0	0	MG372089
OTU 25	0	0	9	1	0	0	0	MG372090
OTU 26	0	0	10	0	5	0	0	MG372091
OTU 27	0	0	31	0	33	0	0	MG372092
OTU 28	0	0	0	20	0	33	0	MG372093
OTU 29	0	0	0	2	0	0	0	MG372094
OTU 30	2	0	993	0	706	0	0	MG372095
OTU 31	0	0	3	0	4	0	0	MG372096
OTU 32	0	7	0	0	0	0	0	MG372097
Total	22486	41626	43110	31111	43048	39059	1	

The scripts used for the different analyses are deposited at https://github.com/douglasgscofield/pubs/tree/master/Brannstrom-et-al-1.

## Results

### DNA extractions, PCR, Sanger and Ion Torrent sequencing

By using PCR and Sanger sequencing, we successfully amplified and sequenced the ITS region of the mycobiont from 29 of the 30 lichen specimens, and the ITS region of the photobiont from all 30. For the Ion Torrent sequencing of the photobionts, DNA was extracted and the ITS1 region was successfully amplified from all six pooled samples (Table 1). None of the negative controls showed amplicons by gel electrophoresis. Only one sequence of ITS per thallus was obtained by Sanger technology, while with Ion Torrent we discovered reads of multiple photobiont OTUs: after delineation and quantification of the photobiont amplicon sequences, 32 OTUs were identified. Each OTU ID together with the number of reads for all OTUs and their prevalence in each sample is shown in Table 2. Sequencing of the negative control resulted in 1 read (Table 2).

### No cross-contamination of mycobionts in the pooled samples

PCR-reactions using *Thamnolia* or *Cetraria* specific primers (Table S1) verified that single genera were found in each pooled tissue-sample, and thus, that the samples were not cross-contaminated prior to the Ion Torrent sequencing step. Specifically, we amplified a PCR product from the *Cetraria* samples when *Cetraria* specific primers were used, but not when *Thamnolia* specific primers were used, and *vice versa*. In contrast, all samples that were mixed in different proportions with DNA originating from both *Cetraria* and *Thamnolia* tested positive with both primer combinations, which suggests that any cross contamination at the level of 10% would have been detected (data not shown).

### Taxonomic identification of the mycobiont using Sanger ITS data

**Figure 2:**
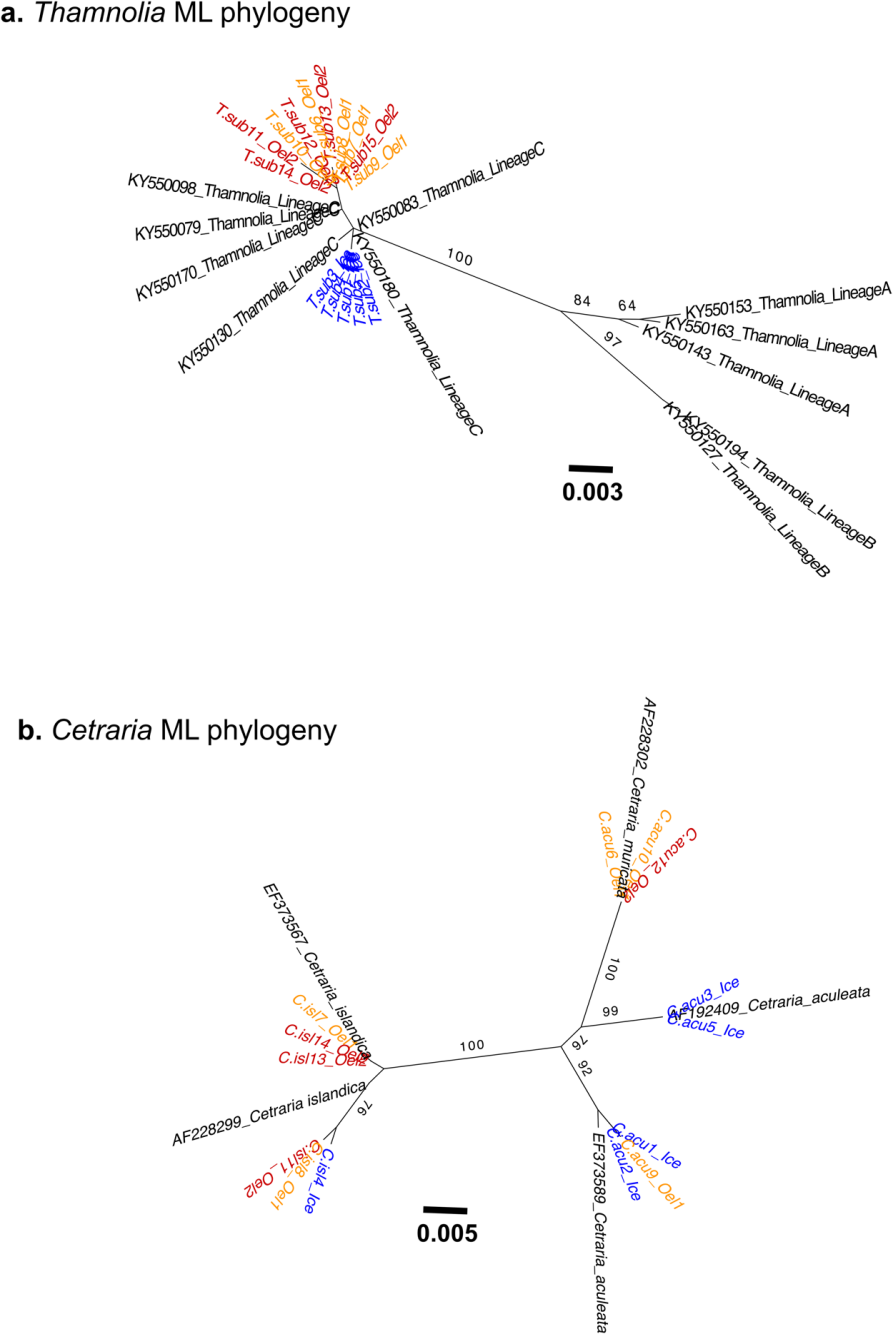
Unrooted mycobiont phylogenies of the *Thamnolia* (**a**) and *Cetraria* (**b**) mycobionts based on the complete ITS region obtained with Sanger sequencing. Our newly generated sequences are highlighted in colours: the samples from Iceland (Ice) in blue; the ones from Öland 1 (Oel1) in yellow and the ones from Öland 2 (Oel2) in red. ‘C.acu’ codes for *Cetraria aculeata*; ‘C.isl’ codes for *Cetraria islandica*, and ‘T.sub’ for *Thamnolia subuliformis*. Numbers from 1 to 15 are given for each *Thamnolia* and *Cetraria* specimen investigated in this study (see Table 1). The accession numbers and the species/lineage name of the sequences retrieved from GenBank are shown in black. The support (<60%) for each branch is given as bootstrap values shown above branches. The scale bar indicates the branch length.

The mycobiont phylogeny based on the Sanger sequences of the ITS region of *Thamnolia* (Fig. 2a) shows that all newly generated sequences group together with *Thamnolia* GenBank sequences of a clade previously identified as *Thamnolia* Lineage C^31^ and subsequently named *Thamnolia subuliformis* s. str.^62^. In contrast, our *Cetraria* phylogeny (Fig. 2b) splits our samples into four supported lineages. Based on the GenBank references sequences, shown in black font in Fig. 2b, all samples identified as *C*. *islandica* group together with the reference of *C*. *islandica*. However, the specimens that were morphologically identified as *C*. *aculeata* are paraphyletic, two groups cluster with *C*. *aculeata* reference and one with the one of *C*. *muricata* (Fig. 2b).

### Taxonomic identification of the photobiont using Sanger and Ion Torrent ITS data

**Figure 3:**
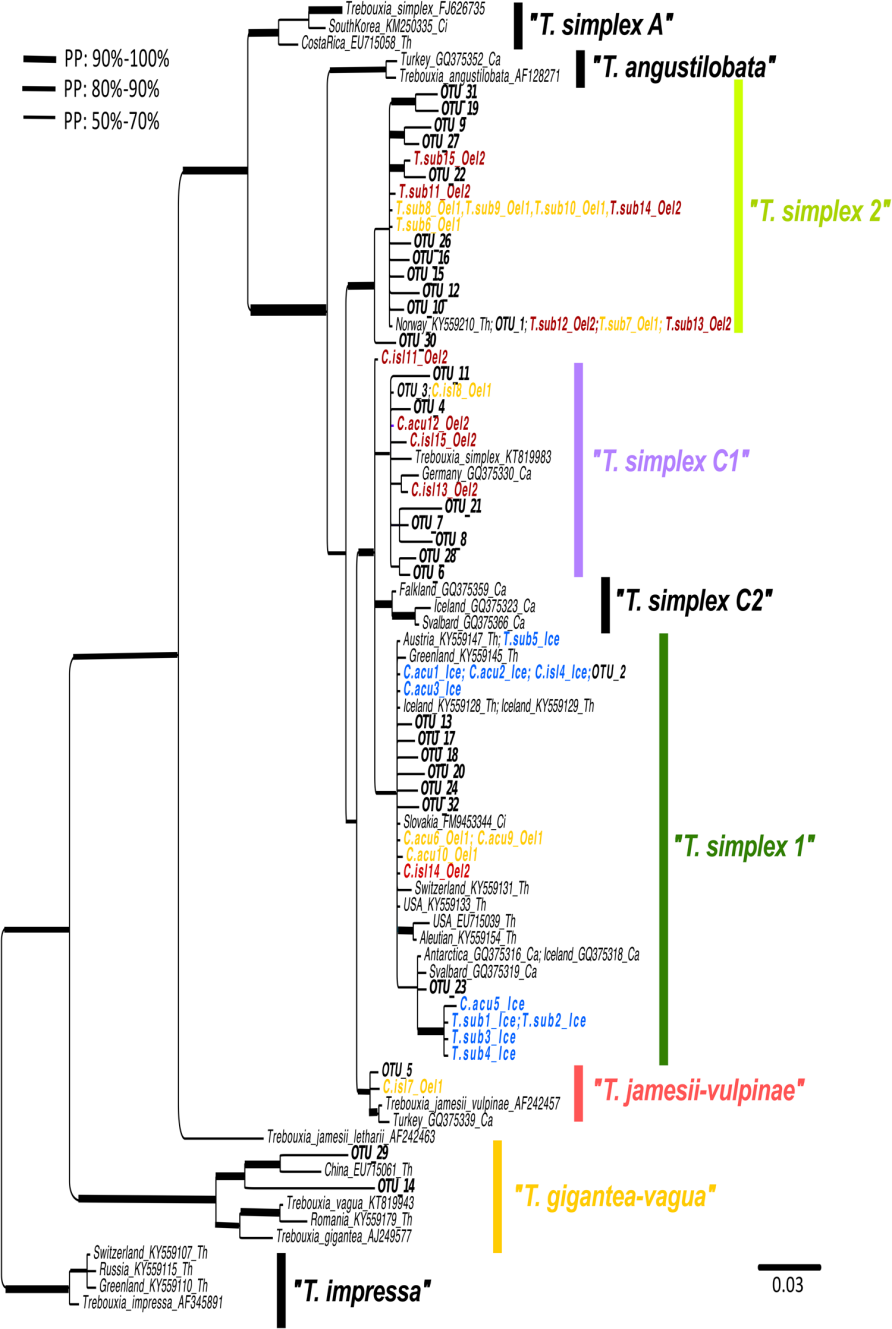
Rooted photobiont phylogeny based on the ITS 1 region, with data were obtained with Sanger and Ion Torrent sequencing technologies. Our newly generated Sanger sequences are highlighted in colors: the samples from Iceland (Ice) in blue; the ones from Öland 1 (Oel1) in yellow and the ones from Öland 2 (Oel2) are in red. ‘C.acu’ codes for *Cetraria aculeata*; ‘C.isl’ codes for *Cetraria islandica*, and ‘T.sub’ for *Thamnolia subuliformis*. Numbers from 1 to 15 are given for each *Thamnolia* and *Cetraria* specimen investigated in this study. The data obtained through Ion Torrent are in bold black and named as OTUs with their identity given as numbers from 1 to 32. The GenBank numbers are shown in black; the accession numbers and the species/lineage name can be seen in the sample ID. The support for each branch (posterior probabilities) is shown by branch thickness. The scale bar indicates the branch length.

In the phylogenetic analyses of the Sanger and Ion Torrent sequences of the ITS region of the photobionts, all 5 MCMC chains converged and were combined in the 50% majority consensus rule tree. The *Trebouxia impressa* clade was used as outgroup. The phylogeny showed that both *Thamnolia* and *Cetraria* mycobionts are associated with several green algae of *Trebouxia*. Specifically, the phylogeny shows 7 well-supported main lineages of *Trebouxia* with posterior probabilities (PP) above 80%. Because of the uncertain species status in *Trebouxia*, the 7 lineages are referred to herein as ‘*T*. *simplex A’*, ‘*T*. *simplex 2′*, *T*. *simplex C’* (further on divided in three other clades: ‘C1’, and ‘C2’), ‘*T*. *angustilobata’*, ‘*T*. *simplex jamesii-vulpinae’*, ‘*T*. *gigantea-vagua’*, and *‘T*. *impressa’* (Fig. 3).

Most of the Ion Torrent sequences were found in four of the clades: ‘*T*. *simplex 2′*, ‘*T*. *simplex C1’*, ‘*T*. *simplex 1′*, and ‘*T*. *jamesii-vulpinae’*. The exceptions were OTU 29 and OTU 14 which groups in the ‘*T*. *gigantean-vagua’* clade together with a photobiont sequence amplified from a *Thamnolia* lichen specimen from China (accession number EU715061: Fig. 3). Notably, this sequence was amplified with Sanger technology by another research group and retrieved by us from GenBank.

Some of the photobiont clades were dominated by sequences retrieved from either *Thamnolia* or *Cetraria*. For example, ‘*T*. *impressa’* and *‘T*. *simplex 2′* clades are dominated by *Thamnolia*’s photobionts, while *T*. *angustilobata’*, ‘*T*. *simplex C2’*, and ‘*T*. *jamesii-vulpinae’* exclusively contain photobionts of *Cetraria* (Fig. 3). On the other hand, the subclade named ‘*T*. *simplex 1′* contains photobiont sequences from both lichen genera, from all three investigated localities (Gardabaer, Öland 1 and Öland 2), but also from other localities of both hemispheres (Fig. 3).

Due to its divergence from the other sequences, OTU 25 was not included in the phylogeny and was identified based on a nucleotide blast search in the NCBI database. The best hit was *Hemichloris antarctica* which had a 100% query cover, an e-value of 1e-37 and an identity score of 80% was (GenBank accession number: HG972970). OTU 25 was found in low abundance in *Cetraria* (Öland 1, with 1 read) and *Thamnolia* (Öland 1, with 9 reads) (Table 2).

### Sharing of photobionts between *Thamnolia* and *Cetraria*

**Figure 4:**
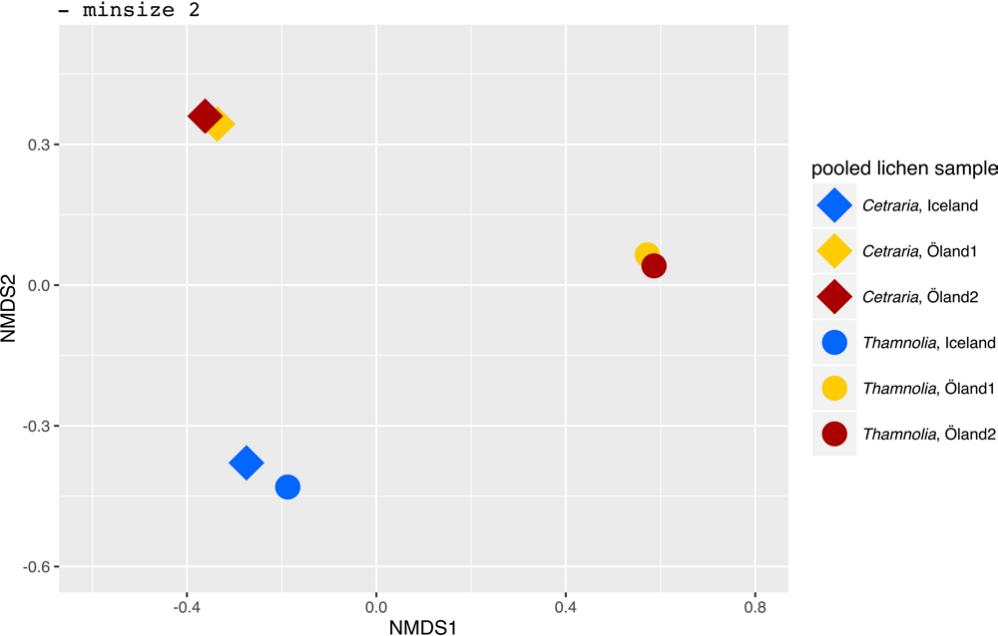
NMDS clustering of the photobiont composition, based on Ion Torrent data, of the six pooled lichen samples: one *Thamnolia* (depicted with circles) and one *Cetraria* (depicted with rhombs) from each of the three localities: Iceland (blue), Öland 1 (yellow) and Öland 2 (red). For all samples the reads were pooled, dereplicated, and clustered into OTUs using the constraint of requiring each OTU to be supported by at least two reads (−minsize 2). The Bray-Curtis dissimilarity was used and the stress value was 1.

**Figure 5:**
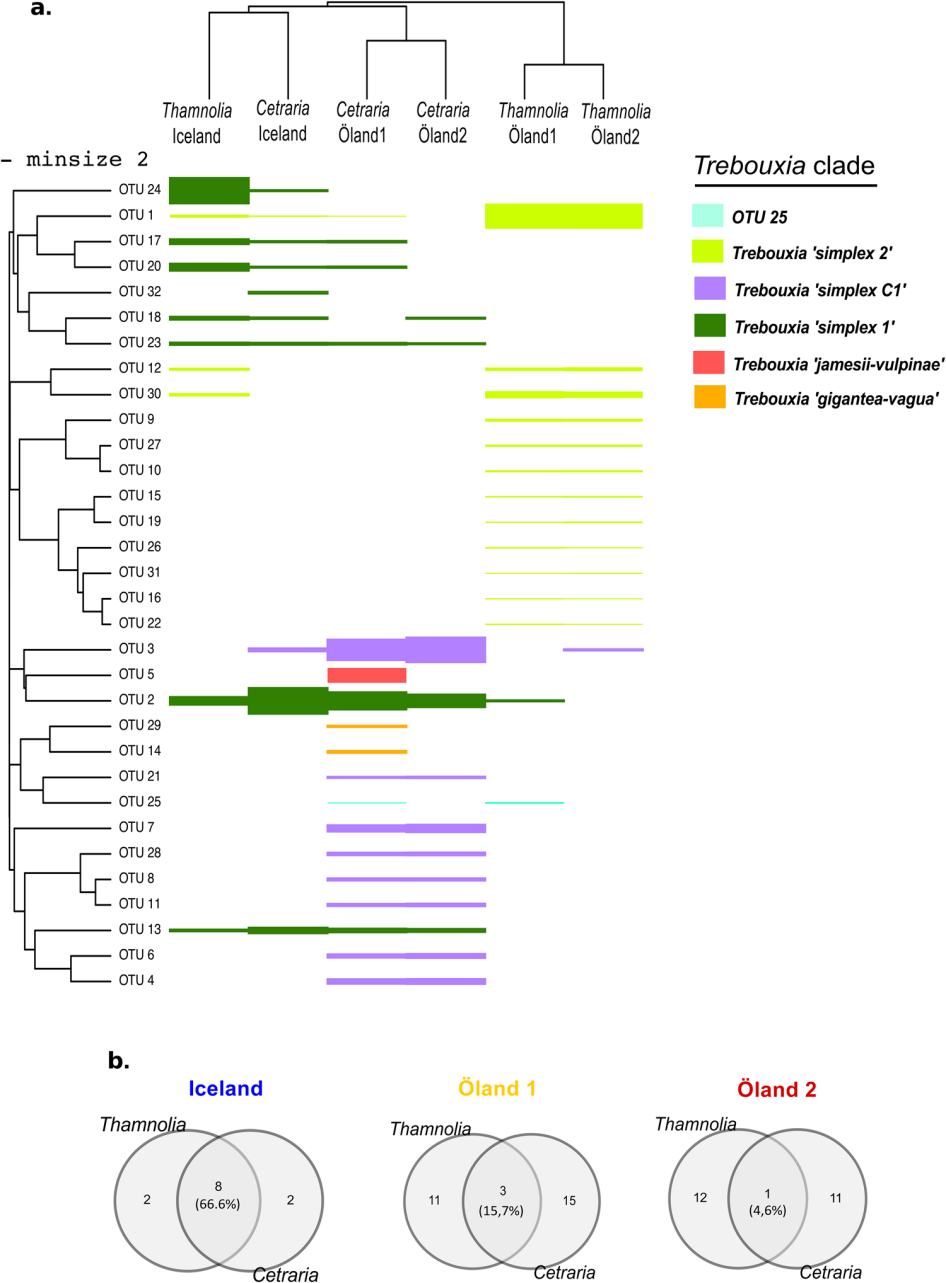
Photobiont diversity within the samples of *Thamnolia* and *Cetraria*, based on Ion Torrent data (**a**) Hierarchical clusters based on photobiont OTU relative quantities. The number of reads for each OTU in each sample is shown by bar thickness, which is scaled via its cube-root for display. The left dendrogram clusters OTUs by similarity in composition and abundances, and the top dendrogram clusters site/genus pairs by similarities in composition and abundances. The colors of the bars reflect the genotypic identity of each OTU based on the ITS1 photobiont phylogeny (Fig. 3). For all samples the reads were pooled, dereplicated, and clustered into OTUs using the constraint of requiring each OTU to be supported by at least two reads (−minsize 2). (**b**) Venn diagrams showing the proportion of shared and exclusive OTUs between *Thamnolia* and *Cetraria* pooled samples within each locality.

Both the Sanger and the Ion Torrent datasets indicate that the photobiont community is more similar between *Thamnolia* and *Cetraria* on Iceland than on Öland. First, the photobiont phylogenetic analyses of the complete ITS region obtained with Sanger technology (see Supplementary Fig. S3) showed that the photobionts of *Thamnolia* and *Cetraria* from Iceland associate with closely related photobionts. Most of the photobionts of *Cetraria* specimens from Öland are closely related with the lichens from Iceland, while the *Thamnolia* photobionts form a separate clade (see Supplementary Fig. S3). The data from Ion Torrent shows the same trend but at higher resolution, since more genotypes were recovered by this analysis. Both clustering analyses of the Ion Torrent data (Figs 4 and 5) together with the phylogenetic analyses of the amplicons of the ITS1 region (Fig. 3) group the photobiont communities of the two lichen genera from Iceland together. The Venn diagrams of Fig. 5b show that circa 67% of the photobiont OTUs are common between *Thamnolia* and *Cetraria* at melur (Iceland). Most of this shared photobiont OTUs belong to a clade identified as *Trebouxia simplex 1* (Fig. 5a), which was also encountered in *Cetraria* specimens from Öland. The Icelandic *Thamnolia* and *Cetraria* were also found associated with a low frequency OTU (OTU 1) that was identified as *Trebouxia simplex 2* (Fig. 5a). Noteworthy, OTU1 was found in high abundance in *Thamnolia* samples from Öland (Fig. 5a; Table 2).

At alvar (Öland 1 and Öland 2), each lichen genera have a high proportion of exclusive OTUs but also few shared ones (ca. 16% for Öland 1 and 5% for Öland 2) (Fig. 5b). Both *Thamnolia* pooled samples from the alvar are predominantly associating with OTUs genotyped as *‘Trebouxia simplex 2′* (Fig. 5a; Table 2). But they also contain two low frequencies OTUs (OTU2 and OTU3) that are found in high proportions in *Cetraria* lichens and they belong to *‘Trebouxia simplex 1′* and *‘Trebouxia simplex C1’*, respectively. *Cetraria* samples from Öland1 were additionally associated with photobiont OTUs belonging to the genotypes of *‘T*. *jamesii-vulpinae’* (OTU 5), *‘T*. *gigantean-vagua’* (OTU14 and OTU29) (Fig. 5a,b) but also *‘Trebouxia simplex 2′* (OTU 1).

These overall results of differential sharing hold when applying more stringent filtering in requiring each OTU to be supported by at least 3, instead of 2 reads (see Supplementary Figs S4 and S5).

## Discussion

### *Thamnolia* and *Cetraria* associate with diverse, and partly overlapping, *Trebouxia* photobionts

Our results confirm the findings of previous studies indicating that mycobionts of *Thamnolia* and *Cetraria* are flexible towards their photobionts in that they can associate with different *Trebouxia* lineages^4,31,39^ (Fig. 3). Although three of these photobiont lineages were detected only in either *Thamnolia* (*Trebouxia ‘impressa’*, Fig. 3) or *Cetraria* (*Trebouxia ‘jamesii-vulpinae’*, *Trebouxia ‘angustilobata’*, Fig. 3), all the other clades contained photobionts retrieved from both lichen genera. Our results were based on observations from three sites, but nevertheless showed that *Thamnolia* and *Cetraria*, which are often found growing together, can harbour an overlapping spectrum of genetically diverse photobionts in their thalli.

It is worth pointing out that we cannot with this study design confirm without doubt that the identified algal OTUs have their origin from within the thalli, and thus, that they represent true photobionts (c.f. refs^36,63^). Other studies, which were specifically interested in investigating the intrathalline photobiont diversity in several lichen species employed a more stringent approach by washing the lichen thalli with different solutions^36,63^. However, the only certain confirmation of the algal origin from within the thalli is the culturing of single cells of intrathalline photobionts^33^, or specific labelling, such as for example the one used in the recent study by Spribille *et al*. 2016^64^. Both of these last two approaches require *a priori* knowledge of the present intra-lichen diversity, which was not available at the onset of our study. The only taxon that appears to be epithallic from our data is OTU 25, which is divergent (*Hemichloris* or similar, i.e., not a known lichen photobiont^38^) and was found in a small fraction of samples only on Öland 1, suggesting that external contamination is not a major issue for our study. Nevertheless, we are left with essentially the same result: sharing of a broad diversity of photobiont taxa, whether endo- or epithallic, which differs between species and sites.

### HTS sequencing suggests that multiple photobiont genotypes exist within lichen thalli

The presence of a single photobiont in a lichen thallus usually found when using PCR and Sanger sequencing of molecular markers (e.g. ref.^25^) could be biologically true for certain lichens, but recent work indicates that it might be an artefact of the traditional Sanger method, which is biased towards producing a predominant genotype as its single amplification product. Accordingly, with Sanger, we identified one genotype per individual thallus investigated herein, while the Ion Torrent data suggests the coexistence of several algal genotypes within single lichen individuals. Because our samples were pooled before Ion Torrent sequencing, we were not able to directly investigate the distribution of genotypes within individual thalli with this data. However, the finding of more photobiont genotypes (10 to 18 OTUs) than the number of studied thalli (5) in each sample suggests the simultaneous presence of multiple photobionts in *Thamnolia* and *Cetraria* lichens. The finding of more OTUs than number of thalli holds also when applying more stringent filtering in requiring each OTU to be supported by at least 3 reads, indicating that this result is not due to, e.g., rare OTUs originating from sequencing errors. Furthermore, we verified the coexistence of multiple photobiont genotypes within single lichen thalli by using specific primers to amplify and Sanger-sequence several *Trebouxia* lineages (‘*T*. *simplex’*, ‘*T*. *impressa’* and ‘*T*. *vagua-gigantea’* – Fig. 3) (data not shown).

The idea that symbiotic organisms are not a homogenous population of autotrophic symbionts but rather a mosaic of local adapted photobiont strains was also shown in some corals systems^65^, and is consistent with several lichen studies that have used other molecular techniques such as restriction fragment polymorphism^37^, microsatellites^34^ or HTS technologies to show that individual thalli contain more than one photobiont genotype^36,66^.

In conclusion, all these recent studies are challenging the paradigm that lichen thalli contain homogeneous photobiont populations and highlight the importance of using more sensitive techniques than PCR and Sanger sequencing to the study of lichen photobionts. For example, if macrolichens such as *Thamnolia* and *Cetraria* contain heterogeneous intrathalline photobiont populations, approaches that reveal only the most abundant genotype (c.f. refs^4,31,39,67^) can give misleading results, e.g., infer photobiont switching and replacement in cases where instead dynamic fluctuations of photobiont within lichen thalli is taking place.

### Photobiont sharing in sympatric populations of *Thamnolia* and *Cetraria*

Data from both sequencing technologies showed convincingly that the photobionts associated with *Thamnolia* and *Cetraria* from Iceland are closely related with each other, and with photobionts from *Cetraria* individuals from Öland (Figs 3 and 5a). Based on these genetic similarities we suggest that on Iceland, *Thamnolia* and *Cetraria* share photobionts, while on Öland the two lichen genera associate with distinct photobiont communities, although few OTUs were shared. Sharing of photobionts between closely related mycobiont species with similar ecologies was previously shown in lichens^23,24^ but rarely described between distantly related mycobiont genera^66^. Together with other studies, which show that lichen communities from harsh climatic conditions share photobionts^20,25,66^, our results support the hypothesis that mycobionts with similar ecological requirements, such as those specific to arctic environments, might share a common pool of photobionts^21,12,17,35^. Furthermore, the presence of similar genotypes across wide geographic ranges, combined with occurrence of multiple photobiont genotypes within lichen thalli, suggest a continuous exchange of photobionts between *Thamnolia* and *Cetraria*, by yet unknown mechanisms.

### The significance of differential photobiont abundance in lichen thalli

If the proportion of reads generated by Ion Torrent sequencing is used to assess the predominant genotype within a lichen, we see that, as an example, on Öland one genotype (OTU3; *T*. *simplex C1*) is dominant in the *Cetraria* thalli, while on Iceland a genetically different photobiont is most abundant in *Cetraria* (OTU2; *T*. *simplex 1*) (Fig. 5a). *Thamnolia* sampled on Iceland also harbours OTUs belonging to *T*. *simplex 1*, but based on the proportion of reads shows more abundantly photobionts of OTU 24. The amount of the different photobiont genotypes in a lichen population may be a result of chance alone, or simply reflect the demographic history and/or the specificity of the mycobiont towards their photobionts^68^. However, one may also hypothesize that the photobionts confer a phenotypic impact on the lichen and that the abundant photobiont provides a fitness advantage of the individual thallus, (*i*.*e*., that the abundance of photobionts is driven by the adaptation to the local environment). Experiments investigating differential fitness of lichen thalli in response to relative abundance of photobionts have to our knowledge not been carried out. However, in line with this hypothesis, when photobiont lineage specific primers have been used in combination with monocultures of photobiont cells from lichen thalli, it has been shown that coexisting algal genotypes with complementary physiological performances^15^ could be found within a lichen individual^33,69^. Such selection of genetically similar but distinct photobionts within a thallus fits within the ‘fungal-farmers hypothesis’, in which mycobionts acquire available photobionts and subsequently select the appropriate genotype as the farming observed in ant growing fungi^19^ or the use of environmentally adapted photobionts in corals^2,70^. Yet another, not mutually exclusive, adaptive model is that the selection is acting on the photobiont, so that it increases in abundance in the thallus, but that this process is independent of the mycobiont.

### The effect of lichen dispersal on photobiont community in thalli

Photobiont sharing between distantly related lichens suggest that horizontal transmission of photobiont occurs^11^ and our data supports this notion. However, the mechanism of photobiont recruitment in macrolichens such as *Thamnolia* and *Cetraria* with a predominantly or even exclusively vegetative dispersal mode is still not understood. Does photobiont switching take place only when fungal spores spread by themselves and resynthesize the lichen by acquiring a photobiont obtained from an external source? Independent dispersal of the symbionts to Iceland and local reassembling of the lichen in that area could explain some of the observed sharing of photobiont populations between *Thamnolia* and *Cetraria* and why *Thamnolia* specimens from Öland seem to contain a different spectrum of photobionts (Fig. 5a). Furthermore, our data of identical, or very similar, photobiont genotypes from different parts of the world (Fig. 3) suggest that the photobiont community is also affected by long-distance codispersal of the symbionts, which is likely to take place by vegetative propagules. One third factor that may affect the photobiont distribution in lichens is the continuous acquisition of photobionts after the lichen is formed, a phenomenon shown to take place in corals^70^, but never proven in lichens. The combination of these routes of lichen dispersal and photobiont acquisition, together with the ability to maintain a diverse set of photobionts within a thallus, may be advantageous for exclusively or predominantly vegetative spreading lichen species to adapt to new environments.

## Conclusion

Our results confirm that lichens may contain heterogeneous populations of photobionts that can be shared between species^15,33–37^. The lichen guild hypothesis postulates that distantly related but sympatric lichenized fungi may share a common photobiont pool^21,24,34^, and it was also shown that geographically separated lichens that occupy similar niches may have similar photobionts^4,12,17,18^. Based on this earlier work, together with our findings of similar genotypes across wide geographic ranges and the occurrence of multiple photobiont genotypes within *Thamnolia* and *Cetraria* thalli, we suggest an extended hypothesis regarding lichens adaption to the environment. We speculate that, just as reef-forming corals^70^, predominantly vegetatively dispersing lichens acquire photobionts during both incipient and adult life stages (idea also proposed in 2010 by Asplund and co-workers^71^). Depending on the environment, the most adapted photobiont increases in relative frequency within the thallus, at the same time as the accumulated intrathalline diversity is retained. Dispersal through vegetative propagules that maintain the photobiont diversity of the thallus will increase the chance for a successful establishment of lichen individuals in the new environments. Additional data from a diverse range of lichen species addressing for example the distribution of photobionts within individual thalli combined with fitness experiments, is needed in order to confirm this adaptive hypothesis and prove its generality in lichen symbiosis.

## Electronic supplementary material

Supplementary material
