# Supplementary material for: Sharing of photobionts in sympatric populations of Thamnolia and Cetraria lichens: evidence from high-throughput sequencing
Source: Sci Rep. 2018 Mar 13;8:4406. doi: 10.1038/s41598-018-22470-y (PMC5849601; doi:10.1038/s41598-018-22470-y)
Supplement: Supplementary file 1 — Supplementary material [file 41598_2018_22470_MOESM1_ESM.pdf]

Supplementary Information

Differential sharing of photobionts in sympatric populations of *Thamnolia* and *Cetraria* lichens: evidence from high-throughput sequencing

Ioana Onuț-Brännström[1], Mitchell Benjamin[1], Douglas G. Scofield[2,3], Starri Heiðmarsson[4], Martin G.I. Andersson[5], Eva S. Lindström[5], Hanna Johannesson[1*]

[1]Systematic Biology, Department of Organismal Biology, Uppsala University, Sweden
[2]Evolutionary Biology, Department of Ecology and Genetics, Uppsala University, Sweden
[3]Uppsala Multidisciplinary Center for Advanced Computational Science (UPPMAX), Uppsala University, Sweden
[4]Icelandic Institute of Natural History, Akureyri Division, Borgir vid Nordurslod, Iceland
[5]Limnology, Department of Ecology and Genetics, Uppsala University, Sweden

Author of correspondence: Hanna Johannesson, tel. +46 18 4716479 email: hanna.johannesson@ebc.uu.se

The following Supporting Information is available for this article:
Fig. S1 Sanger and Ion Torrent sequencing treatments.
Fig. S2 Rarefaction curves for OTU richness estimates for all samples
Fig. S3 Unrooted mycobiont phylogeny based on the complete ITS region.
Fig. S4 NMDS clustering of the photobiont composition.
Fig. S5 Hierarchical clusters based on photobiont OTU relative quantities (`-minsize 3`).
Table S1 Primers used in this study.
Table S2 Photobiont GenBank Sanger sequences used in the study.

Fig. S1 Sanger and Ion Torrent sequencing treatments. For each selected specimen, we divided the lichen tissue in two halves: one half to be analyzed using Sanger sequencing and the other one with Ion Torrent technology. For Sanger sequencing, DNA extractions were separately performed on each specimen. For Ion Torrent sequencing we pooled for each locality the five specimens from *Thamnolia* into one sample, and the five specimens of *Cetraria* in another sample.

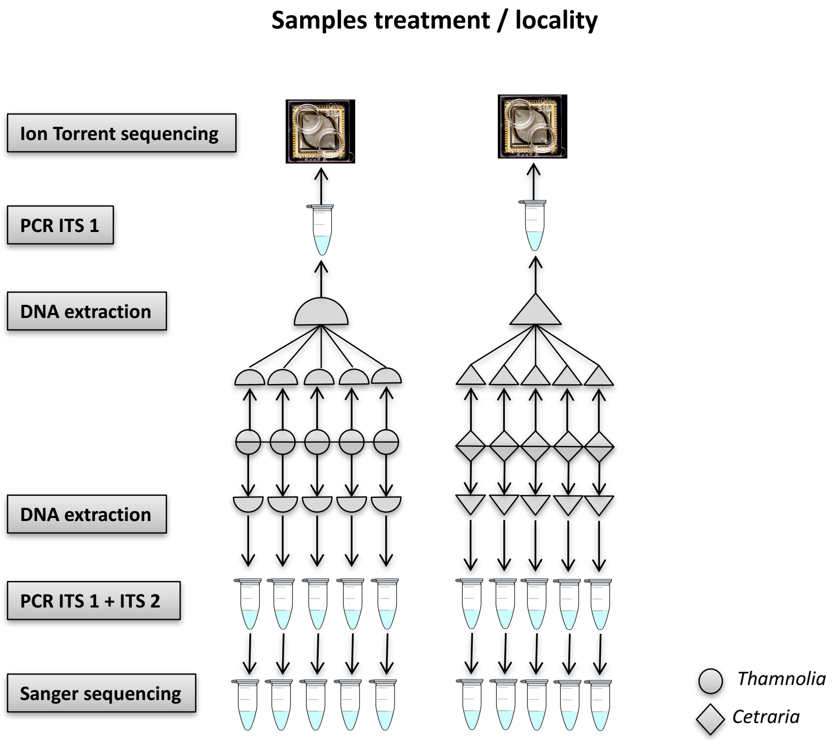

Fig. S2 Rarefaction curves for OTU richness estimates for all samples. The rarefaction curves were estimated for *Trebouxia* OTU richness estimates for all samples, together with observed values. Rarefaction was performed with `estimated_observation_richness.py` from QIIME 1.9.1 (see main text for references) using rarefaction sample sizes from N = 1 to N = 44971.

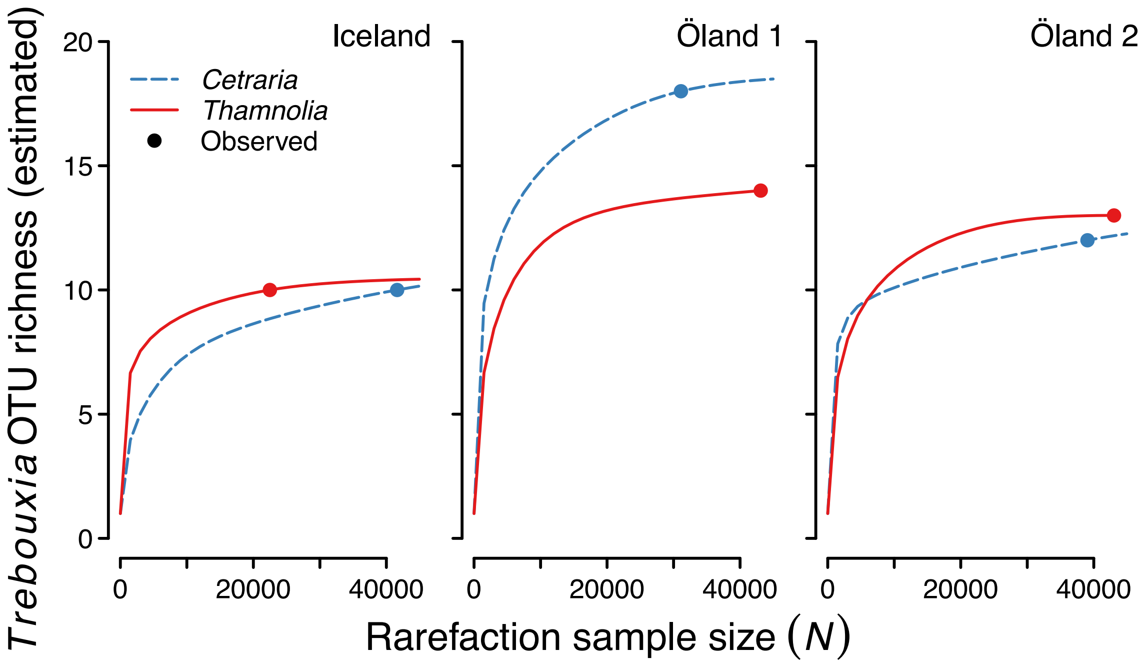

Fig. S3 Unrooted mycobiont phylogeny based on the complete ITS region. The data were obtained with Sanger sequences. The colors indicate the origin of each sample: Iceland (Ice) in blue; Öland 1 (Oel1) in yellow and Öland 2 (Oel2) in red. 'C.acu' codes for *Cetraria aculeata*; 'C.isl' codes for *Cetraria islandica*, and 'T.sub' for *Thamnolia subuliformis*. Numbers from 1 to 15 are given for each *Thamnolia* and *Cetraria* specimen investigated in this study. The support for each branch is given as bootstrap values and is shown above branches. The branch length is indicated by the scale bar.

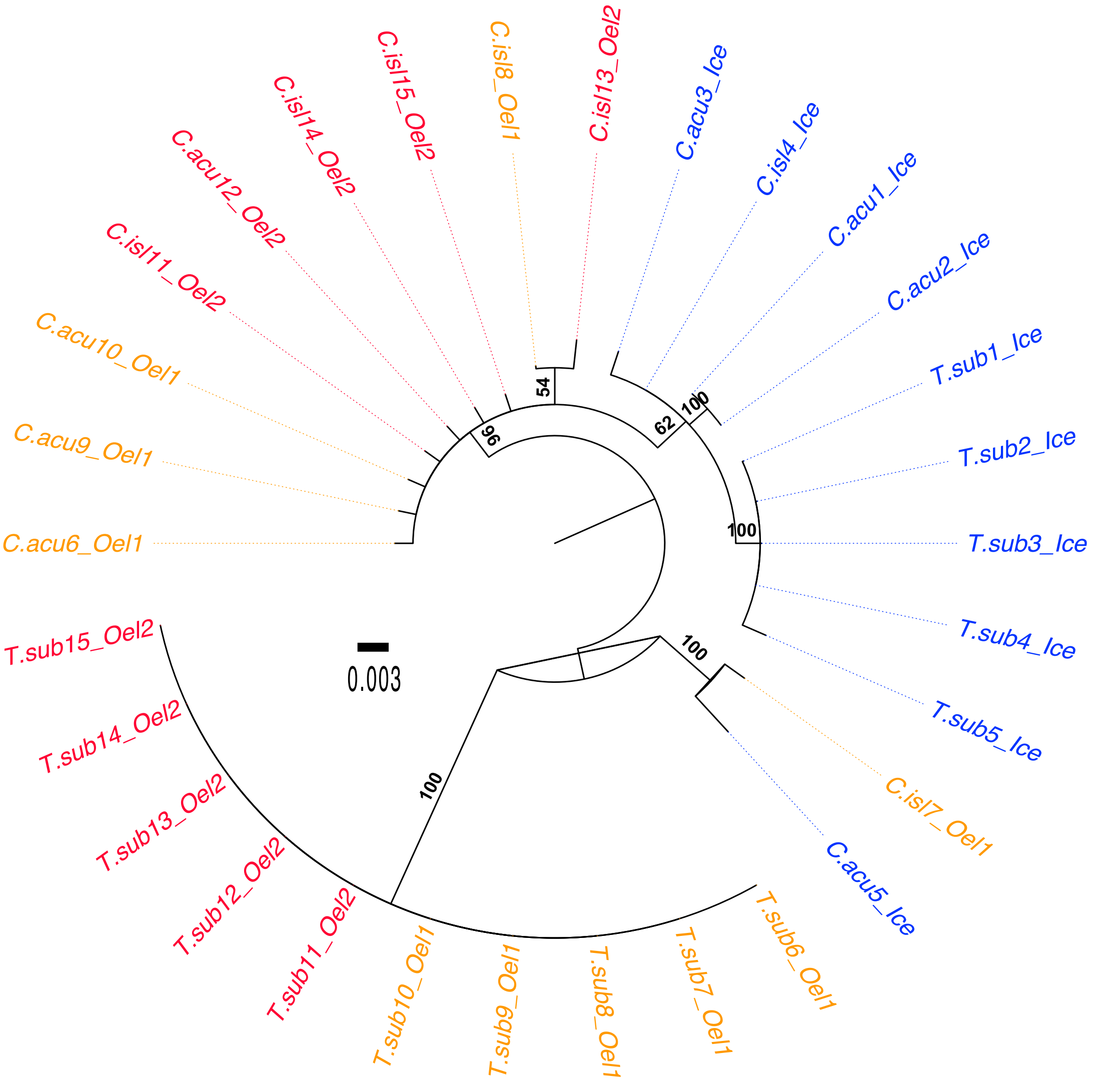

Fig. S4 NMDS clustering of the photobiont composition. The clustering was based on Ion Torrent data, of the six pooled lichen samples: one *Thamnolia* (depicted with circles) and one *Cetraria* (depicted with rhombs) from each of the three localities: Iceland (blue), Öland 1 (yellow) and Öland 2 (red). For all samples the reads were pooled, dereplicated, and clustered into OTUs using the constraint of requiring each OTU to be supported by at least three reads (−`minsize 3`). The Bray-Curtis dissimilarity was used and the stress value was 1.

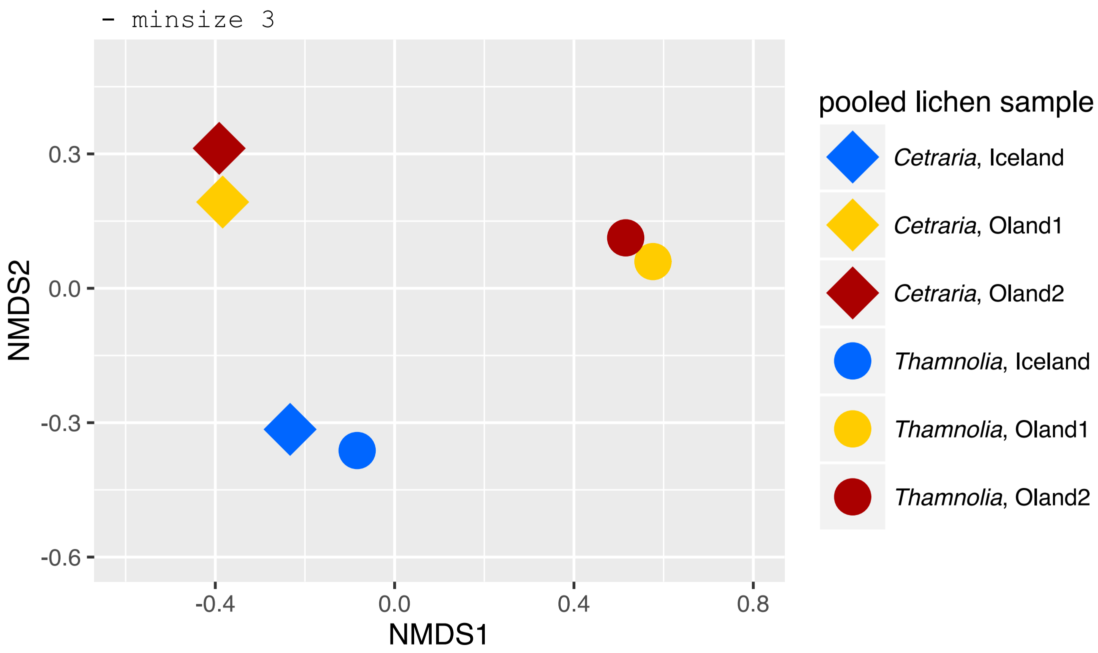

Fig. S5 Hierarchical clusters based on photobiont OTU relative quantities (`-minsize 3`). The number of reads for each OTU in each sample is shown by bar thickness, which is scaled via its cube-root for display. The left dendrogram clusters OTUs by similarity in composition and abundances, and the top dendrogram clusters site/genus pairs by similarities in composition and abundances. For all samples the reads were pooled, dereplicated, and clustered into OTUs using the constraint of requiring each OTU to be supported by at least three reads (`-minsize 3`).

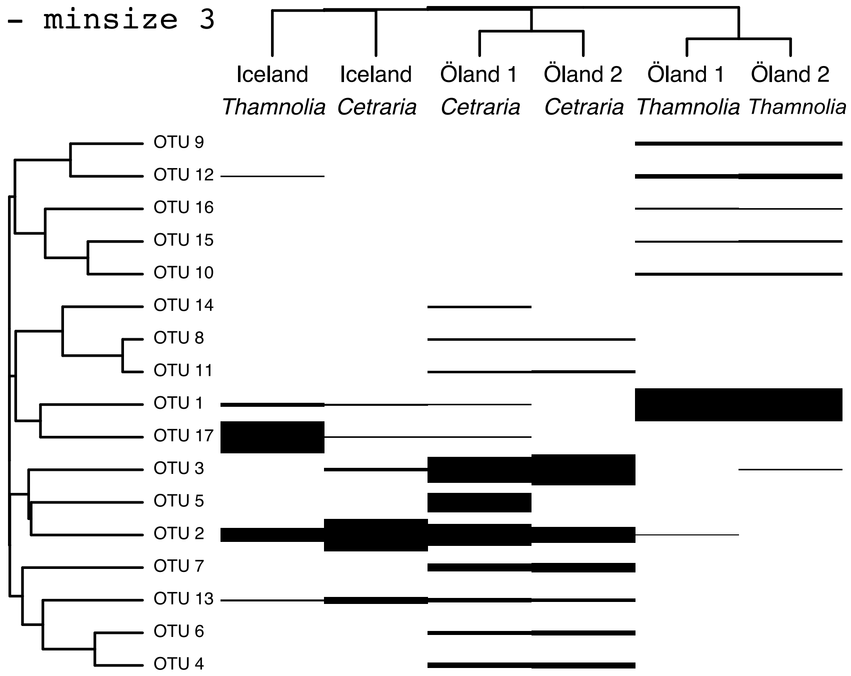

Table S1. Primers used in this study.

Primer name	Primer Sequence 3' -5'	direction	Sequencing technology	References	Targeted symbiont
ITS1F	CTTGGTCATTTAGAGGAAGTAA	forward	Sanger	Gardes & Bruns, 1993	mycobiont, general primers
ITS4	TCCTCCGCTTATTGATATGC	reverse	Sanger	Gardes & Bruns, 1994	mycobiont, general primers
ITS_CeGen_F60	TGTACGGGGTTRTCTGGC	reverse	Sanger	primers designed for this study	mycobiont specific Cetraria.
ITS_CeGen_R491	MCCCTTTGTGTACCAACCT	forward	Sanger	primers designed for this study	mycobiont specific Cetraria
ITS_Th_Rev790	TCACAGTCCAATTTCTCAAAGG	forward	Sanger	primers designed for this study	mycobiont specific Thamnolia
Th_anc_R1	CGTATCCTGGGACTTAGAGC	reverse	Sanger	primers designed for this study	mycobiont specific Thamnolia
ITS1T	GAAGGATCATTGAATCTATCGT	forward	Sanger	Kroken & Taylor, 2000	photobiont, general primers
ITS4T	GGTTCGCTCGCCGCTACTA	reverse	Sanger	Kroken & Taylor, 2000	photobiont, general primers
LG_ITSa_F1	CCATCTCATCCCTGCGTGTCTCCGACTCAGCTAAGGTAACGATGAAGGATCATTGAATCTATCGT	forward	Ion Torrent	primers designed for this study	photobiont, barcoded primers
LG_ITSa_F2	CCATCTCATCCCTGCGTGTCTCCGACTCAGTAAGGAGAACGATGAAGGATCATTGAATCTATCGT	forward	Ion Torrent	primers designed for this study	photobiont, barcoded primers
LG_ITSa_F3	CCATCTCATCCCTGCGTGTCTCCGACTCAGAAGAGGATTCGATGAAGGATCATTGAATCTATCGT	forward	Ion Torrent	primers designed for this study	photobiont, barcoded primers
LG_ITSa_F4	CCATCTCATCCCTGCGTGTCTCCGACTCAGTACCAAGATCGATGAAGGATCATTGAATCTATCGT	forward	Ion Torrent	primers designed for this study	photobiont, barcoded primers
LG_ITSa_F5	CCATCTCATCCCTGCGTGTCTCCGACTCAGCAGAAGGAACGATGAAGGATCATTGAATCTATCGT	forward	Ion Torrent	primers designed for this study	photobiont, barcoded primers
LG_ITSa_F6	CCATCTCATCCCTGCGTGTCTCCGACTCAGCTGCAAGTTCGATGAAGGATCATTGAATCTATCGT	forward	Ion Torrent	primers designed for this study	photobiont, barcoded primers
LG_ITSa_F7	CCATCTCATCCCTGCGTGTCTCCGACTCAGTTCGTGATTCGATGAAGGATCATTGAATCTATCGT	forward	Ion Torrent	primers designed for this study	photobiont, barcoded primers
LG_ITSa_F8	CCATCTCATCCCTGCGTGTCTCCGACTCAGTTCCGATAACGATGAAGGATCATTGAATCTATCGT	forward	Ion Torrent	primers designed for this study	photobiont, barcoded primers
LG_ITSa_R1	CCTCTCTATGGGCAGTCGGTGATTTCGCTGCGTTCTTCATCGTT	reverse	Ion Torrent	primers designed for this study	photobiont, barcoded primers

Table S2. Photobiont GenBank Sanger sequences used in the study. For the photobionts amplified from *Thamnolia* or *Cetraria* the country and the GenBank number can be seen in the sequence ID given for each sample. For reference sequences that are used for identification, the *Trebouxia* species and the GenBank number is given in the Sample ID. When known, the lichen amplification source is given for each sample.

#	sequence ID	lichen source
1	Antarctica_GQ375316_Ca	*Cetraria aculeata*
2	Austria_KY559147_Th	*Thamnolia sp.*
3	China_EU715061_Th	*Thamnolia sp.*
4	CostaRica_EU715058_Th	*Thamnolia sp.*
5	Falkland_GQ375359_Ca	*Cetraria aculeata*
6	USA_EU715039_Th	*Thamnolia sp.*
7	Germany_GQ375330_Ca	*Cetraria aculeata*
8	Greenland_KY559110_Th	*Thamnolia sp.*
9	Greenland_KY559145_Th	*Thamnolia sp.*
10	Iceland_GQ375323_Ca	*Cetraria aculeata*
11	Iceland_KY559128_Th	*Thamnolia sp.*
12	Iceland_KY559129_Th	*Thamnolia sp.*
13	Iceland_GQ375318_Ca	*Cetraria aculeata*
14	Norway_KY559210_Th	*Thamnolia sp.*
15	Svalbard_GQ375366_Ca	*Cetraria aculeata*
16	Svalbard_GQ375319_Ca	*Cetraria aculeata*
17	Romania_KY559179_Th	*Thamnolia sp.*
18	Russia_KY559115_Th	*Thamnolia sp.*
19	Slovakia_FM945344_Ci	*Cetraria islandica*
20	SouthKorea_KM250335_Ci	*Cetraria islandica*
21	Switzerland_KY559107_Th	*Thamnolia sp.*
22	Switzerland_KY559131_Th	*Thamnolia sp.*
23	Trebouxia_angustilobata_AF128271	*Lecidea lapicida*
24	Trebouxia_gigantea_AJ249577	*Caloplaca cerina*
25	Trebouxia_impressa_AF345891	*Physcia stellaris*
26	Trebouxia_jamesii_letharii_AF242463	*Letharia lucida*
27	Trebouxia_jamesii_vulpinae_AF242457	*Letharia vulpina*
28	Trebouxia_simplex_FJ626735	unknown
29	Trebouxia_simplex_KT819983	unknown
30	Trebouxia_vagua_KT819943	*Umbilicaria grisea*
31	Turkey_GQ375339_Ca	*Cetraria aculeata*
32	Turkey_GQ375352_Ca	*Cetraria aculeata*
33	Aleutian_KY559154_Th	*Thamnolia sp.*
34	USA_KY559133_Th	*Thamnolia sp.*

Th = *Thamnolia*; Ca = *Cetraria aculeata*; Ci = *Cetraria islandica*
